# Media Coverage, Journal Press Releases and Editorials Associated with Randomized and Observational Studies in High-Impact Medical Journals: A Cohort Study

**DOI:** 10.1371/journal.pone.0145294

**Published:** 2015-12-23

**Authors:** Michael T. M. Wang, Mark J. Bolland, Greg Gamble, Andrew Grey

**Affiliations:** Department of Medicine, University of Auckland, Private Bag 92019, Auckland, New Zealand; Georgia Regents University, UNITED STATES

## Abstract

**Background:**

Publication of clinical research findings in prominent journals influences health beliefs and medical practice, in part by engendering news coverage. Randomized controlled trials (RCTs) should be most influential in guiding clinical practice. We determined whether study design of clinical research published in high-impact journals influences media coverage.

**Methods and Findings:**

We compared the incidence and amount of media coverage of RCTs with that of observational studies published in the top 7 medical journals between 1 January 2013 and 31 March 2013. We specifically assessed media coverage of the most rigorous RCTs, those with >1000 participants that reported ‘hard’ outcomes. There was no difference between RCTs and observational studies in coverage by major newspapers or news agencies, or in total number of news stories generated (all P>0.63). Large RCTs reporting ‘hard’ outcomes did not generate more news coverage than small RCTs that reported surrogate outcomes and observational studies (all P>0.32). RCTs were more likely than observational studies to attract a journal editorial (70% vs 46%, P = 0.003), but less likely to be the subject of a journal press release (17% vs 50%, P<0.001). Large RCTs that reported ‘hard’ outcomes did not attract an editorial more frequently than other studies (61% vs 58%, P>0.99), nor were they more likely to be the subject of a journal press release (14% vs 38%, P = 0.14).

**Conclusions:**

The design of clinical studies whose results are published in high-impact medical journals is not associated with the likelihood or amount of ensuing news coverage.

## Introduction

Randomized controlled trials (RCTs), particularly those that are large and assess ‘hard’ outcomes, provide the most rigorous evidence to guide clinical practice [1]. In contrast, observational studies can generate hypotheses but not reliably test them [2]. However, observational research is conducted more frequently than randomized studies, and both types of research are published in prominent medical journals. Thus, both types of research potentially influence health beliefs and behaviours.

Publication of clinical research findings in prominent medical journals is often accompanied by media coverage, including that by outlets with large circulations. Such publications can also be accompanied by an editorial or a journal press release, each of which potentially increases the visibility and impact of the source article. Press releases from journals and academic institutions strongly influence the content of news stories in the lay media about the source research [3–5]. Media coverage of clinical research influences the public’s health knowledge, beliefs and behaviours [6,7]. Evidence that media coverage also influences the behaviours and beliefs of medical scientists [6] suggests that it also affects health practitioners’ behaviours.

There is increasing interest in the effects of media coverage on the impact of health research [8] and in improving the reporting of research findings (http://www.healthnewsreview.org/), but few studies have examined the relationships between research publications and news stories. A recent report suggested that newspapers in the United States with high readerships preferentially cover observational studies rather than randomized trials, and favour reporting of lower quality observational studies [9]. In the current work, we investigated whether study design influences media coverage of articles published in major internal medicine journals. We specifically assessed whether research that should be highly influential in guiding clinical practice, large randomized trials with hard outcomes, was more likely to obtain news coverage than other studies.

## Methods

### Source documents

We searched MEDLINE to collate studies with clinical outcomes published between 1/1/2013 and 3/31/2013 in the New England Journal of Medicine (NEJM), Journal of the American Medical Association (JAMA), Lancet, PLoS Medicine, JAMA Internal Medicine, British Medical Journal (BMJ) and Annals of Internal Medicine. We included meta-analyses of either randomized or observational research, but excluded meta-analyses that evaluated both types of study. We used journal websites and Eurekalert! (www.eurakalert.org) to collate editorials and journal press releases that accompanied the publication of each source article. We used Factiva (https://global.factiva.com) to identify news stories that were generated in response to each article within 2 months of its publication. Factiva accesses more than 30,000 information sources, including newspapers, journals, magazines, and television and radio transcripts. We assessed all news stories associated with a publication, and separately assessed news stories reported in the top 10 USA and UK newspapers by circulation (http://auditedmedia.com/news/blog/top-25-us-newspapers-for-march-2013.aspx; http://www.abc.org.uk/Certificates-Reports/Our-Reports/, accessed June 2015) and the top 10 English language news agencies by website traffic (http://www.alexa.com/topsites/category/Top/News. accessed June 2015). The latter includes both print and audiovisual media.

### Data extraction

From each source publication, we extracted data on journal of publication, study design, sample size, study duration, outcomes assessed and study results. We applied the Institute of Medicine definition of surrogate outcomes as “biomarker[s] intended to substitute for a clinical endpoint [and] expected to predict clinical benefit (or harm…) based on epidemiologic, therapeutic, pathophysiologic, or other scientific evidence” [10]. “Hard” outcomes are patient-important endpoints that are definitive with respect to the disease process, and reflect how a patient feels, functions or survives [10,11]. We compared categorical variables between observational and randomized studies using Fisher’s exact tests or chi-squared tests. The total number of news stories generated from each study was analysed as a continuous, non-normally distributed variable and compared between observational and randomized studies using Kruskal-Wallis tests, in Prism version 6.05. Confidence intervals for proportions were calculated using OPENEPI (www.openepi.com). P<0.05 was considered significant.

## Results

We assessed 171 source articles that reported 86 observational studies and 85 RCTs, 100 accompanying editorials and 584 news stories (S1 Table). NEJM does not issue press releases, so the 44 journal press releases we assessed were generated for 126 articles that reported 70 observational studies and 56 RCTs. Table 1 shows the characteristics of the studies, and the journals of publication. Of the 584 news stories, 167 (29%) were published in one of the top 10 newspapers, and 120 (21%) were reported by one of the top 10 news agencies.

**Table 1:** Characteristics and journal of publication of clinical studies.

	Observational studies (N = 86)	Randomized controlled trials (N = 85)
**Journal of publication,** N (%)		
Annals of Internal Medicine	8 (9)	3 (4)
BMJ	24 (28)	13 (15)
JAMA	10 (12)	11 (13)
JAMA Internal Medicine	13 (15)	3 (4)
Lancet	8 (9)	25 (29)
NEJM	16 (19)	29 (34)
PLOS Medicine	7 (8)	1 (1)
**“Hard” primary outcome,** N (%)	77 (90)	38 (45)
**Sample size > 1000,** N (%)	68 (79)	36 (42)
**Study duration, y** median (95% CI)	3.0 (0.9–5.0)	1.0 (0.5–1.0)
**Statistically significant effect on primary outcome,** N (%)	65 (76)	54 (64)

**Fig 1:**
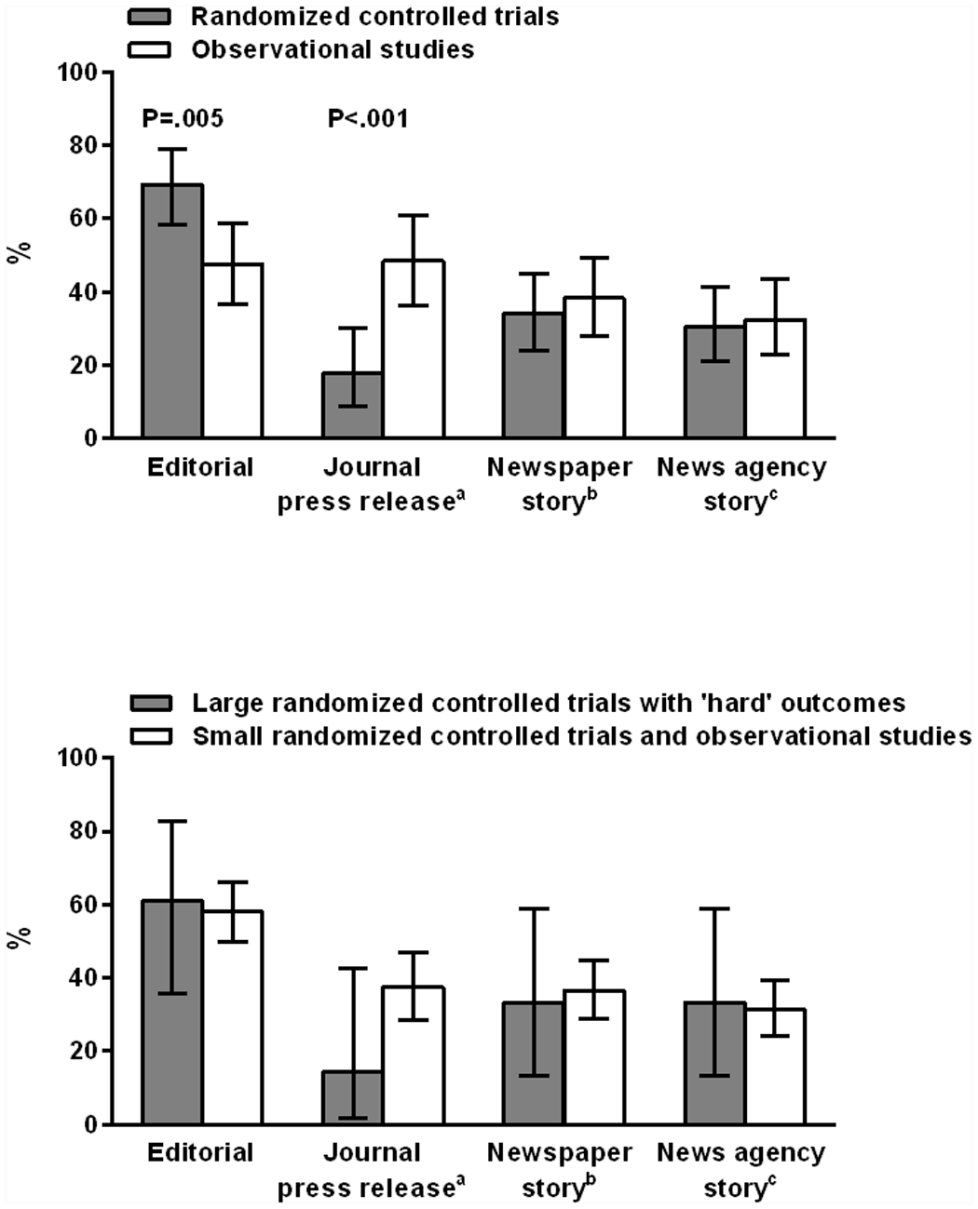
Associations between study design, journal documents and news stories. *Top panel*, the proportions of the indicated documents associated with 86 observational studies (open bars) and 85 randomized controlled trials (shaded bars) published in major internal medicine journals. *Lower panel*, the proportions of the indicated documents associated with 153 small randomized controlled trials and observational studies (open bars) and 18 large randomized controlled trials with “hard” outcomes (shaded bars) published in major internal medicine journals. Horizontal bars indicate bounds of 95% CI.. a, The New England Journal of Medicine does not issue press releases, so the number of evaluable observational studies for this analysis was 70, and of RCTs was 56. b, news story reported in at least 1 of the top 10 print newspapers, by circulation; The Sun, Daily Mail, Daily Mirror, Evening Standard, Daily Telegraph, Daily Star (UK); Wall Street Journal, New York Times, USA Today, Los Angeles Times (USA). c, news story reported by at least 1 of the top 10 English language news agencies, by website traffic; CNN, New York Times, The Guardian, Times of India, Fox News, BBC News, Washington Post, Wall Street Journal, USA Today, Reuters.

Observational studies were less likely than RCTs to attract an accompanying editorial (41 of 86, 48%, vs 59 of 85, 69%, P = .005) but more likely than RCTs to generate a journal press release (34 of 70, 49%, vs 10 of 56, 18%, P < .001) (Fig 1, top panel). Media coverage did not differ by study design. Coverage by at least 1 major newspaper occurred for 33 of 86 (38%) observational studies and 29 of 85 (34%) RCTs (P = .63). Coverage by at least 1 major news agency occurred for 28 of 86 (33%) observational studies and 26 of 85 (31%) RCTs (P = .87). Considering all media coverage, the median (95% CI) number of news stories generated per study was 2 (1–3) for observational studies and 1 (1–2) for RCTs (P = .81).

18 source articles reported large (>1000 participants) RCTs with “hard” outcomes. Journal and media coverage of these trials did not differ from that generated in response to the 153 observational studies and RCTs with smaller samples size and/or surrogate endpoints (Fig 1, lower panel). Eleven of 18 large RCTs (61%) with “hard” outcomes were accompanied by editorials, compared to 89 of 153 (59%) of other studies (P>0.99): journal press releases were issued for 2 of 14 (14%) of the large RCTs with “hard” outcomes, compared to 42 of 112 (38%) of other studies (P = 0.14). Six of the 18 (33%) large RCTs with “hard” outcomes received coverage in a major newspaper, and 6 of 18 (33%) received coverage from a major news agency: for other studies the respective proportions were 56 of 153 (37%, P>0.99) and 48 of 153 (31%, P>0.99). The median (95% CI) number of news stories generated per study for the large RCTs with “hard” outcomes was 1 (1–2), similar to that generated in response to the other studies, which was 1 (1–3), (P = 0.31).

## Discussion

RCTs represent a higher level of evidence than observational studies [1]. Consequently, it might be expected that academic commentary and media coverage would occur more frequently for randomized research than observational research. We found that editorials in high-impact journals were more commonly written for RCTs than observational studies. However, journal press releases, which influence the content of subsequent news stories [4,5], were more common for observational studies than RCTs. The occurrence and amount of media coverage was similar for observational studies and RCTs, both overall and when coverage by major newspapers or news agencies was specifically assessed. Large RCTs that report “hard” disease outcomes should be very influential on practice but, in comparison to studies with less rigorous design or importance, they were not more frequently accompanied by editorials or journal press releases, nor did they generate more media coverage.

Our results complement those of Selvaraj et al, who compared the design of studies reported in newspapers with those published in high impact journals [9]. They found that newspapers were more likely to cover observational studies and less likely to cover RCTs than high impact journals, and selected observational research of low quality for coverage. We focused on research most likely to attract news coverage because it was published in a major journal and found that studies with weaker designs were as likely to attract media coverage as those with robust designs.

The preferential reporting of observational research might have important clinical implications, by having undue influence in shaping health practices. Analyses of existing clinical practices that are subsequently contradicted by large RCTs indicate that many become established as a result of observational research [12,13]. The potential for promotion of practices based on weak observational evidence is heightened by the propensity of authors of observational studies to make clinical practice recommendations based on their work [14], and by inadequate reporting of the important limitations of observational studies by researchers, journals and news media [15,16].

Our study has limitations. We only analysed publications in journals with high impact factors: reporting of research published in other journals might be different. Only 18 of the 85 RCTs assessed met our definition of larger trials with ‘hard’ outcomes, reducing the statistical power for comparisons with studies with weaker designs. Our analyses were limited to English language media outlets, and to news stories published within 2 months of publication of the source article.

In summary, we found that the study design of clinical studies published in high impact medical journals is not associated with the likelihood or amount of ensuing news coverage. To improve the dissemination of research evidence, news stories should focus on research that is most capable of accurately informing clinical practice. Journals could assist this process by issuing more press releases that highlight results from randomized trials.

## Supporting Information

S1 Table Data on characteristics of source articles, and associated news stories, journal press releases and editorials. (XLSX)
